# The Genus *Carissa*: An Ethnopharmacological, Phytochemical and Pharmacological Review

**DOI:** 10.1007/s13659-017-0123-0

**Published:** 2017-02-27

**Authors:** Joseph Sakah Kaunda, Ying-Jun Zhang

**Affiliations:** 1 State Key Laboratory of Phytochemistry and Plant Resources in West China, Kunming Institute of Botany, Chinese Academy of Sciences, Kunming, 650201 People’s Republic of China; 2 Graduate School of the Chinese Academy of Sciences, Beijing, 100039 People’s Republic of China; 3 Yunnan Key Laboratory of Natural Medicinal Chemistry, Kunming Institute of Botany, Chinese Academy of Sciences, Kunming, 650201 People’s Republic of China

**Keywords:** *Carissa*, Apocynaceae, Ethnomedicine, Phytochemistry, Triterpenes, Nortrachelogenin, Pharmacology

## Abstract

*Carissa* L. is a genus of the family Apocynaceae, with about 36 species as evergreen shrubs or small trees native to tropical and subtropical regions of Africa, Asia and Oceania. Most of *Carissa* plants have been employed and utilized in traditional medicine for various ailments, such as headache, chest complains, rheumatism, oedema, gonorrhoea, syphilis, rabies. So far, only nine *Carissa* species have been phytochemically studied, which led to the identification of 123 compounds including terpenes, flavonoids, lignans, sterols, simple phenolic compounds, fatty acids and esters, and so on. Pharmacological studies on *Carissa* species have also indicated various bioactive potentials. This review covers the peer-reviewed articles between 1954 and 2016, retrieved from Pubmed, ScienceDirect, SciFinder, Wikipedia and Baidu, using “Carissa” as search term (“all fields”) and with no specific time frame set for search. Fifteen important medicinal or ornamental *Carissa* species were selected and summarized on their botanical characteristics, geographical distribution, traditional uses, phytochemistry, and pharmacological activities.

## Introduction

*Carissa* L., a genus of the family Apocynaceae with about 250 genera, consists of 36 species as evergreen shrubs or small trees native to tropical and subtropical regions of Africa, Asia and Oceania [1, 2]. Among which, four species, including two introduced plants, *C. carandas* L. and *C. macrocarpa* (Eckl.) A. DC., are distributed in China [3]. *Carissa* species possess handsome, glossy foliage and fragrant, starry-white, jasmine-like flowers. The fruits are ornamental and edible, scarlet to crimson in color, oval in shape and are produced after flowering [2]. Numerous *Carissa* plants have been employed and utilized in traditional medicine for various ailments, such as headache, chest complains, rheumatism, oedema, gonorrhoea, syphilis, rabies. They have been also used as a remedy for fever, sickle cell anaemia, cough, ulcer, toothache, and worm infestation [1]. So far, only nine *Carissa* species have been phytochemically studied. Terpenes [4–31], flavonoids [5, 6, 19, 32, 33], lignans [5, 9, 19, 26, 34–36], sterols [5, 6, 11, 15, 17, 31, 37, 38], simple phenolic compounds [5, 6, 9, 13, 14, 32, 36, 39], esters [6, 17, 21], fatty acids [17, 40] and other compounds [5–7, 9, 17, 20, 21, 32, 34] were identified across different species. Pharmacological studies on *Carissa* species have indicated significant antiplasmodial [5, 41], diuretic [42], anticonvulsant [43], antibacterial [9, 13, 19, 44, 45], anti-oxidant and anti-tumor [14, 21, 24, 46–49], antiviral [50–52], antiemetic [53], anti-hyperlipidemic [54], analgesic, anti-inflammatory, antipyretic activities [55–57], vasorelaxant [58], cardioprotective [59], hepatoprotective [38, 60–62], antidiabetic [63] and antihelminthiasis activities [64, 65].

The present article puts forward 15 important medicinal or ornamental *Carissa* species and reports on their botanical characteristics, geographical distribution, traditional uses, isolated chemical constituents, structural illustrations and their investigated pharmacological activities.

## Species’ Description, Distribution and Traditional Uses

Fifteen species used mostly as important folk medicine, ornamental plants, or wild food resources were selected, and their local names, botanical description, distribution and traditional uses were summarized in Table 1.

**Table 1:** Local names, botanical description, distribution and uses of *Carissa* species

Scientific names	Local names	Distribution	Uses
*C. bispinosa*	Num-num (English), Noemnoem (Afrikaans)	Southwestern parts of Western Cape along coastal areas, Eastern Cape, KwaZulu-Natal, Gauteng, Northern provinces, Eastern Free State, Lesotho, Swaziland, Zimbabwe, Mozambique, Botswana, Namibia, Kenya [2, 66]	Ornamental, berries to make jams and jellies, roots treat toothache [2]
*C. boiviniana*		Madagascar [66]	Unknown
*C. carandas*	“Crane berry” (English), *karonda* (Devanagari), Karonda (Hindi), Karonda, Karmard (Sanskrit), Kalakai (Tamil), Vakkay, Peddakalavi (Telgu), Karakka (Malayalam), Karjige Gujarati, Karamdaa (Kannada), Karvinda (Marathi), Karamcha (Bengali), Ci-Huang-Guo (Chinese)	Himalayas, Siwalik Hills, Western Ghats, Nepal, Afghanistan, India, Myanmar, Sri Lanka, Indonesia, China [3, 60], Himalayas, Siwalik Hills, Western Ghats, Nepal, Afghanistan, India, Myanmar, Sri Lanka, Indonesia, China [3, 60]	Antihyperglycemic, hepato-protective [38, 60–62], colic, rheumatoid arthritis, piles, indigestion, splenomegaly, anorexia, cardiac diseases, oedema, amenorrhoea, anti-emetic, cardiotonic, anti-bacterial [44, 45], anti-inflammatory, analgesic and anti-pyretic [55, 56], helminthiasis [64, 65], constipation and diarrhea [67], purgative, snake bite antidote and remittent fever [68, 69]
*C. congesta*	Karamcha, Karamya, Karancha (Bengali), Karaunda (English)	India, Myanmar, Sri Lanka, Philippines [70]	Fly repellant, sweet ripe fruit for puddings and jellies. Syrup is drunk. Leaves, tussar silk-worm fodder. Wood for fuel, fruits for tanning and dyeing. Unripe fruits as astringent. Ripe fruit for biliousness. Leaf decoction for fever, diarrhea, oral inflammation and earache. Root decoction employed as bitter stomachic and itches. Ornamental [70]
*C. edulis*	Simple spined Num-Num	Botswana, Namibia, Uganda, Cameroon, Eritrea, Ethiopia, Ghana, Guinea, Kenya, Nigeria, Saudi Arabia, Senegal, South Africa, Sudan, Tanzania, Thailand, Vietnam, Cambodia, Myanmar, Japan, Yemen Asia, Indo-China [5]	Antiplasmodial [4, 41], diuretic activities [42], anticonvulsant [43], antiherpetic [50], antiviral [51, 52], antidiabetic [63]
*C. grandiflora*	Natal Plum, common Carissa	Southern Africa (Kwa-Zulu/Natal) [71]	A screen or hedge [71]
*C. haematocarpa*		South Africa-Western Cape to Grahams Town Eastern Cape, Southern regions of Namibia, arid Karoo, semi-Karoo regions [72]	Attracts bees, butterflies, other insects and birds. For boundary and hedge [72]
*C. lanceolata*	Conkerberry (English)	Western and Northern Australia, Queensland [1]	Toothache, respiratory infection, colds, flu, and cleaning of sores [1]
*C. macrocarpa*	Amatungulu (Zulu), Noem-Noem (Afrikaans), Dahua-Jiahuci (Chinese)	Saudi Arabia, South Africa, Uganda, China [3, 18]	Antibacterial, fruit, edible, made into pies, jams, jellies, and sauces. Ornamental and fencing [18]
*C. opaca*		Northern hilly areas of Pakistan, Abbottabad, Murree, Margalla Hills, Kashmir, India, Myanmar, Sri Lanka [73]	Fencing, edible ripe berries. Fruits and leaves for jaundice, hepatitis, asthma and fever; root powder treat wounds and injuries [73]
*C. ovata*	Currant Bush, Lime, Kunkerberry, native scrub, Blackberry, Ulorin, Karey (Australia and Queensland)	Western Australia, south-wards to northeastern New South Wales. Grows in 0–900 m altitude, open or monsoon forest [74, 75]	Ripe fruit edible [74], unripe fruit is poisonous [75]
*C. pichoniana*		Madagascar [66]	Unknown
*C. spinarum*	Indian names: *vaka*, *kalivi*, *kalli* (Andhra Pradesh), *karamacha* (Bengal), *karmarda* (Gujarat), *karekayi*, *garji*, *kavali* (Karnataka), *karavada*, *karanda*, *karwant* (Maharashtra), *karondhu*, *garna*, *kharnu* (Himachal Pradesh), *karunda* (Hindi), *karamarda*, *avighna* (Sanskrit), *kalakkay*, *kalachedi* (Tamilnadu), Jiahuci (Chinese)	Tropical Africa, Southern Asia, dry, sandy, rocky soils of India, Ceylon, Myanmar, Thailand, China [3, 66, 76]	Purgative, rheumatism [57], antidote to snakebites [68, 69], cardiotonic, anticonvulsant, hepatoprotective, antiarthritic, antibacterial, lowering blood pressure [77], cleaning worm infested wounds [78] and chronic joint pains management [79]
*C. tetramera*	Sand num-num	Kenya, Mozambique, Tanzania, Swaziland, South Africa, Zimbabwe [66]	Unknown
*C. yunnanensis*	Yunnan-Jiahuci (Chinese)	China, Sri Lanka [80]	Unknown

## Chemical Constituents

From the genus *Carissa*, a total of 123 compounds have been isolated from nine different species, e.g., *C. bispinosa*, *C. carandas*, *C. congesta*, *C. edulis*, *C. grandiflora*, *C. lanceolata*, *C. macrocarpa*, *C. opaca*, and *C. spinarum*. The compounds comprise triterpenes (**1**–**22**), cardiac glycosides (**23**–**24**), sesquiterpenes (**25**–**40**), monoterpenes (**41**–**59**), flavonoids (**60**–**66**), lignans (**67**–**80**), sterols (**81**–**89**), simple phenolic compounds (**90**–**105**), fatty acids and esters (**106**–**114**), and other kinds of compounds (**115**–**123**), as shown in Table 2.

**Table 2:** Chemical constituents’ classification and trends of distribution in *Carissa* plants

Nos.	Compounds	Plant sources	Parts	References
**1**	Lupeol	*C. carandas*	Root	[14, 15, 31]
		*C. carandas*	Fruit	[16]
		*C. opaca*	Root	[6]
		*C. congesta*	Root	[17]
**2**	16*β*-Hydroxybetulinic acid	*C. carandas*	Root	[31]
**3**	Lupa-12,20(29)-dien-3*β*,28-diol	*C. carandas*	Root	[22]
**4**	Lupeol *β*-hydroxyoctadecanoate	*C. opaca*	Aerial	[26]
**5**	3*β*,27-Dihydroxylup-12-ene	*C. opaca*	Aerial	[26]
**6**	Ursolic acid	*C. carandas*	Root	[14, 15]
		*C. macrocarpa*	Leaf	[18]
		*C. spinarum*	Leaf	[19]
		*C. bispinosa*	Leaf	[20]
**7**	Urs-12-ene-3*β*,22*β*-diol	*C. carandas*	Root	[22]
**8**	Me ursolate	*C. carandas*	Root	[15]
**9**	*α*-Amyrin	*C. carandas*	Root	[31]
**10**	Carissic acid	*C. carandas*	Leaf	[25]
**11**	Carissic acid methyl ester	*C. carandas*	Leaf	[25]
**12**	Carissic acid monoacetate	*C. carandas*	Leaf	[25]
**13**	Carissol	*C. carandas*	Fruit	[27]
**14**	13,27-Cyclosuran-3-one	*C. congesta*	Root	[17]
**15**	Oleanolic acid	*C. carandas* *C. macrocarpa*	Root Fruit	[14] [18]
**16**	*β*-Amyrin	*C. macrocarpa* *C. edulis*	Fruit Leaf	[18] [5]
**17**	Me oleanolate	*C. macrocarpa* *C. lanceolata*	Fruit Stem	[18] [1]
**18**	3*β*-Hydroxyolean-11-en-28,13*β*-olide	*C. macrocarpa*	Fruit	[18]
**19**	Friedours-7-en-3-one	*C. congesta*	Root	[17]
**20**	Arjunolic acid	*C. opaca*	Aerial	[26]
**21**	Carandinol	*C. carandas*	Leaf	[24]
**22**	Betulinic acid	*C. carandas*	Leaf	[24]
**23**	Evomonoside	*C. spinarum*	Root	[28, 34]
**24**	Odoroside H	*C. spinarum* *C. lanceolata* *C. ovata*	Root Root Root	[34] [29] [29]
**25**	Carindone	*C. carandas* *C. lanceolata*	Root Stem	[23] [1]
**26**	(+)-Carissone	*C. edulis* *C. congesta* *C. opaca* *C. lanceolata* *C. carandas*	Root Root Root Stem/root Root	[8–10] [11] [12] [1, 12, 29] [31]
**27**	2*α*-Carissanol	*C. edulis*	Root	[8, 10]
**28**	6*α*-Carissanol	*C. edulis*	Root	[8, 10]
		*C. edulis*	Root	[8]
**29**	(+)-6*β*-Carissanol	*C. edulis*	Root	[8, 10]
**30**	(±)-Aristolone (sesquiterpene)	*C. opaca*	Root	[13]
**31**	4-*Epi*-aubergenone	*C. edulis*	Root	[10]
**32**	Dehydrocarissone	*C. edulis* *C. lanceolata*	Root Stem	[10] [1]
**33**	(+)-*β*-Eudesmol	*C. edulis*	Flower Root	[4, 8] [10]
**34**	Cryptomeridiol	*C. edulis*	Root	[8, 10]
**35**	2(*S*),3,4,4a,5,6-hexahydro-2-(1-hydroxy-1-methylethyl)-4a(*R*),8-dimethyl-1,7-naphthalenedione	*C. edulis*	Root	[8]
**36**	Germacrenone	*C. edulis* *C. spinarum*	Root Stem	[8] [21]
**37**	2,3,3-Trimethyl-2-(3-methylbuta-1,3-dienyl)-6-methylenecyclohexanone	*C. opaca*	Root	[6]
**38**	Zicrone	*C. opaca*	Root	[13]
**39**	Nerolidol	*C. carandas* *C. grandiflora*	Flower Flower	[7] [7]
**40**	Farnesol	*C. carandas* *C. grandiflora*	Flower Flower	[7] [7]
**41**	3-Carene	*C. carandas* *C. grandiflora*	Flower Flower	[7] [7]
**42**	Pinene	*C. edulis*	Root	[4]
**43**	Myrcene	*C. edulis*	Root	[4]
**44**	Limonene	*C. edulis*	Root	[4]
		*C. opaca*	Root	[6]
**45**	Sabanene	*C. edulis*	Root	[4]
**46**	Camphene	*C. carandas* *C. grandiflora*	Flower Flower	[7] [7]
**47**	Menthol	*C. carandas*	Flower	[7]
**48**	*p*-Cymene	*C. carandas* *C. grandiflora*	Flower Flower	[7] [7]
**49**	*α*-Terpineol	*C. carandas* *C. grandiflora*	Flower Flower	[7] [7]
**50**	Piperitone	*C. carandas* *C. grandiflora*	Flower Flower	[7] [7]
**51**	Citronellal	*C. carandas*	Flower	[7]
**52**	Linalyl acetate	*C. grandiflora*	Flower	[7]
**53**	(±)-Linalool	*C. carandas*	Flower	[7]
**54**	Neryl acetate	*C. carandas*	Flower	[7]
**55**	Geranyl acetate	*C. carandas*	Flower	[7]
**56**	*β*-Ionone	*C. carandas*	Flower	[7]
**57**	*γ*-Terpenene	*C. grandiflora*	Flower	[7]
**58**	Geraniol	*C. grandiflora*	Flower	[7]
**59**	2-Isopropenyl-5-methyl-6-hepten-1-ol	*C. congesta*	Root	[17]
**60**	Rutin	*C. edulis* *C. carandas* *C. opaca* *C. congesta*	Leaf Fruit Root Root	[5] [32] [6] [33]
**61**	Epicatechin	*C. carandas*	Fruit	[32]
**62**	Epicatechin gallate	*C. edulis* *C. carandas*	Leaf Fruit	[5] [32]
**63**	Quercetin	*C. carandas* *C. opaca* *C. congesta*	Fruit Root Root	[32] [6] [33]
**64**	Kaempferol	*C. carandas*	Fruit	[32]
**65**	Naringin	*C. spinarum*	Leaf	[19]
**66**	3,5-Dihydroxy-3′,4′,7-trimethoxy-flavone 3-*β*-d-glucoside	*C. edulis*	Leaf	[5]
**67**	Secoisolariciresinol	*C. spinarum* *C. edulis*	Stem Root	[21, 34] [9]
**68**	Carrisanol	*C. spinarum* *C. lanceolata* *C. opaca*	Stem Stem Aerial	[21, 34] [19] [26]
**69**	1,2,4-Butanetriol, 2,3-bis[[4-dimethoxyphenyl)methyl]-,1,4-diacetate	*C. carandas*	Root	[35]
**70**	Carinol	*C. spinarum* *C. carandas* *C. lanceolata* *C. edulis* *C. opaca*	Stem Root Root Root Aerial	[21, 34] [35] [36] [9] [26]
**71**	4,4′-Dimethylcarinol	*C. carandas*	Root	[35]
**72**	1,2,4-Butanetriol, 2,3**-**bis[[4-(acetyloxy)-3-methoxyphenyl] methyl]-,1,4-diacetate	*C. carandas*	Root	[35]
**73**	Lariciresinol	*C. edulis*	Root	[9]
**74**	9′-*O*-methyl-(8*R*,8′*R*,9′*S*)-carrisanol	*C. edulis*	Root	[9]
**75**	9′-*O*-methyl-(8*R*,8′*R*,9′*R*)-carrisanol	*C. edulis*	Root	[9]
**76**	3-(4-Methoxyphenyl)-2,6-dimethyl-benzofuran	*C. opaca*	Root	[13]
**77**	(−)-Olivil	*C. edulis*	Root	[9, 21]
**78**	(+)-Nortrachelogenin	*C. edulis* *C. spinarum*	Root Stem	[5, 9] [21, 34]
**79**	8-Hydroxypinoresinol	*C. spinarum*	Stem	[34]
**80**	(+)-Pinoresinol	*C. spinarum* *C. opaca*	Stem Aerial	[21, 34] [26]
**81**	*β*-Sitosterol	*C. edulis* *C. opaca* *C. carandas*	Leaf Root Root	[5] [6] [15, 31]
**82**	Sitosterol glucoside	*C. edulis* *C. congesta* *C. carandas*	Leaf Root Root	[5] [11] [31]
**83**	Campesterol	*C. spinarum*	Root	[37]
**84**	Cholest-5-en-3*β*-ol	*C. carandas*	Root	[38]
**85**	Stigmasterol glucoside	*C. edulis*	Leaf	[5]
**86**	Stigmasterol	*C. spinarum*	Root	[37]
**87**	3*β*,5*α*-Stigma-7,25-dien-3-ol	*C. congesta*	Root	[17]
**88**	3*β*,5*α*-Stigma-7,16-dien-3-ol	*C. congesta*	Root	[17]
**89**	Chrondrillasterol	*C. congesta*	Root	[17]
**90**	Piceatannol	*C. carandas*	Fruit	[32]
**91**	Resveratrol	*C. carandas*	Fruit	[32]
**92**	Syringic acid	*C. carandas*	Fruit	[32]
**93**	Vanillic acid	*C. carandas*	Fruit	[32]
**94**	Vanillin	*C. opaca* *C. edulis*	Root Root	[6] [9]
**95**	3,4′-Dihydroxypropiophenone	*C. edulis*	Root	[9]
**96**	Coniferaldehyde	*C. spinarum*	Stem	[21, 34]
**97**	*p*-Coumaric acid	*C. carandas*	Fruit	[32]
**98**	Caffeic acid methyl ester	*C. edulis*	Leaf	[5]
**99**	Caffeic acid	*C. carandas* *C. spinarum*	Fruit Root	[32] [39]
**100**	Ellagic acid	*C. carandas*	Fruit	[32]
**101**	2-Acetylphenol	*C. edulis* *C. lanceolata*	Root Root	[9] [36]
**102**	Chlorogenic acid	*C. carandas*	Fruit	[32]
**103**	Chlorogenic acid-1-methylester-1-ethylether	*C. edulis*	Leaf	[5]
**104**	Scopoletin	*C. edulis* *C. carandas* *C. opaca*	Root Root Root	[9] [14] [13]
**105**	Isofraxidin	*C. edulis*	Root	[9]
**106**	Eicosanoic acid	*C. carandas*	Seed	[40]
**107**	Hexadecanoic acid	*C. carandas*	Seed	[40]
**108**	Octadecanoic acid	*C. carandas*	Seed	[40]
**109**	9*Z*,12*Z*-octadecadienoic acid	*C. carandas*	Seed	[40]
**110**	9*Z*-octadecenoic acid	*C. carandas*	Seed	[40]
**111**	3′-(4″-Methoxyphenyl)-3′-oxo-propionyl hexadecanoate	*C. spinarum*	Stem	[21]
**112**	Hexadecanoic acid 2-hydroxyl-1-(hydroxymethyl) ethyl ether	*C. congesta*	Root	[17]
**113**	Butyl-9,12-octadecadienoate	*C. congesta*	Root	[17]
**114**	2-Benzenedicarboxylic acid, mono(2-ethylhexyl) ester	*C. opaca*	Root	[6]
**115**	l-Ascorbic acid	*C. carandas*	Fruit	[32]
**116**	Dihydrojasmone	*C. carandas*	Flower	[7]
**117**	4-Amino-1-(4-amino-2-oxo-1(2*H*)-pyrimidinyl)-1,4-dideoxy-*β*-d-glucopyranuronic acid	*C. carandas*	Root	[30]
**118**	6-Decaprenylphenol	*C. carandas*	Root	[30]
**119**	9-Octadecene	*C. congesta*	Root	[17]
**120**	Tritriacontane	*C. bispinosa*	Leaf	[20]
**121**	Vitamin E	*C. opaca*	Root	[6]
**122**	Naphthalenone	*C. opaca*	Root	[4]
**123**	Pinitol	*C. edulis*	Leaf	[5]

### Triterpenes

**Fig. 1:**
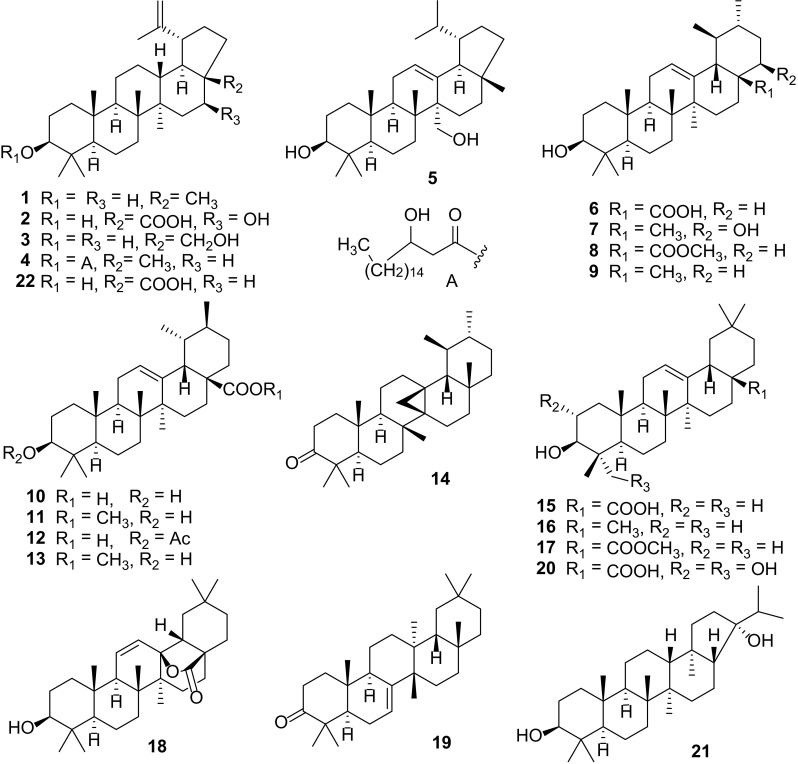
Triterpenes **1–22** from *Carissa*

Twenty-two compounds referring to lupane (**1**–**5**, **22**), ursane (**6**–**14**), oleanane (**15**–**18** and **20**), D:C-friedoleane triterpene (**19**) and isohopane (2**1**) type triterpene (Fig. 1), have been isolated mostly from the roots of *C. carandas* [14, 15, 25, 27, 31], *C. congesta* [17], *C. lanceolata* [29], *C. opaca* [6, 26], and *C. spinarum* [28, 34]. It showed that pentacyclic oleanane triterpenes, oleanolic acid (**15**), *β*-amyrin (**16**), methyl oleanolate (**17**) and ursane triterpene, ursolic acid (**6**), had been isolated mostly from the roots of *C. carandas* [14, 15] and the aerial parts of *C. macrocarpa* [18]. Isolation of ursolic acid (**6**) had also been achieved from the leaves of *C. spinarum* [19] and *C. bispinosa* [20]. Other triterpenoids, lupeol *β*-hydroxyoctadecanoate (**4**) and 3*β*,27-dihydroxylup-12-ene (**5**) had also been isolated and characterized by Parveen S. et al. from the aerial parts of *C. opaca* [26].

Galipali S. et al. investigated the anti-inflammatory potential of root methanol extracts of *C. carandas* using bioassay guided fractionation of extract based on inhibitory potential towards proinflammatory mediators [TNF-a, IL-1b and nitric oxide (NO)]. They found out that lupeol (**1**) and oleanolic acid (**15)** exhibited potential anti-inflammatory activities [56].

Carandinol (**21**) was isolated from the leaves of *C. carandas*, along with three known triterpenoid acids, ursolic acid (**6**), oleanolic acid (**15**), and betulinic acid (**22**), and its structure as 3*β*,21α-dihydroxyisohopane was deduced by exhaustive spectroscopic analyses [24]. In the same investigation, carandinol (**21**) was evaluated for cytotoxicity, immunomodulatory, antiglycation, anti-oxidant and enzyme inhibition activity. It exhibited significant in vitro cytotoxicity to every cell line tested (HeLa, PC-3 and 3T3) and was relatively more toxic to human cervical cancer (HeLa) cell line. Their study was the first to report the isolation of a cytotoxic isohopane triterpene, carandinol (**21**), from the genus *Carissa*. Considering the highest number of triterpenes isolated and their trends of distribution across the species, there is a higher probability that they are the most predominant constituents of *Carissa.*

### Cardiac Glycosides

**Fig. 2:**
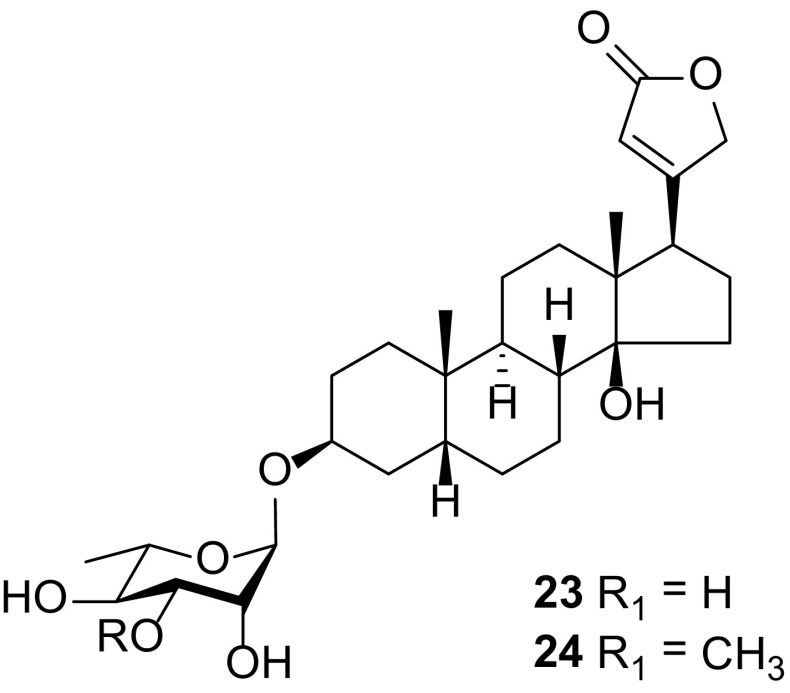
Cardiac glycosides **23** and **24** from *Carissa*

Cardiac glycosides (Fig. 2, compounds 23 and 24) are compounds that occur naturally in certain plants species. They possess qualities that have effects on the heart, stomach, intestines, and nervous system. Just as the name cardiac suggests, these compounds are the active ingredient in many different heart medicines in clinical use and they are the major class of medications used to treat heart failure. The cardiotonic activity of *C. edulis* and its ability to lower blood pressure has been previously reported to be attributed to the presence of the odoroside glucosides, odorosides H (**24**) and F [10].

In an effort by Wangteeraprasert R. et al. to find new antiherpetic agents from the stems of *C. spinarum* [34], the cardiac glycoside evomonoside (**23**) was found to be the only antiherpetic principle, showing moderate activity against herpes simplex virus (HSV) types I and II in the inactivation method [34].

So far, only two cardiac glycosides, evomonoside (**23**) and odoroside H (**24**) have previously been identified from the roots of *C. spinarum* [28, 34]. Odoroside H (**24**) was also isolated from the roots of *C. lanceolata* [29]. Mohr K. et al. reported that the roots of *C. ovata* also contain a little odoroside H [29] (Fig. 2).

### Sesquiterpenes

**Fig. 3:**
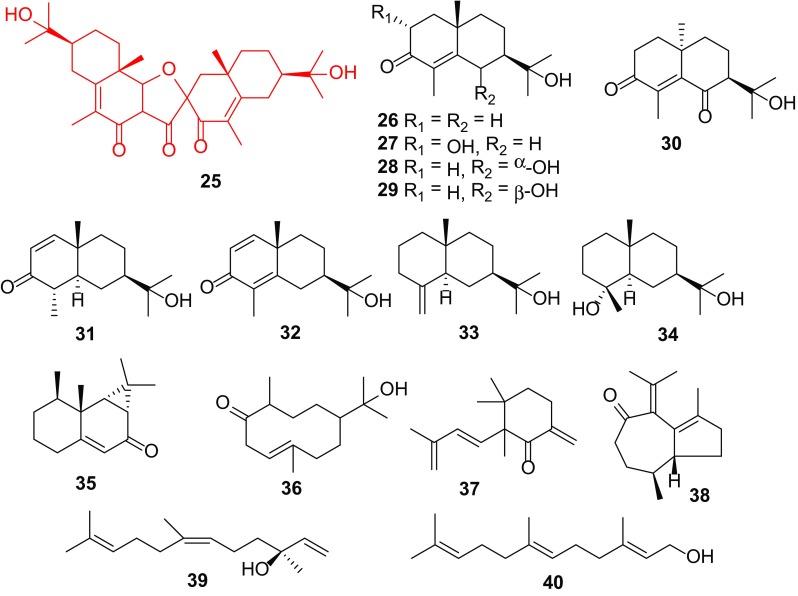
Sesquiterpenes **25–40** from *Carissa*

Sesquiterpenes of *Carissa* have been shown to possess antimicrobial, antimalarial, anticancer and anti-inflammatory effects [10]. Sixteen compounds **25**–**40** have been isolated from seven *Carissa* species (Table 2; Fig. 3). They have been identified from the roots of *C. edulis* [7, 8], *C*. *congesta* [11], *C*. *opaca* [13], *C*. *lanceolata* [1, 12, 29], and the flowers of *C. grandiflora* and *C. carandas* [6]. They comprise 10 sesquiterpenes cyclized into two adjoining cyclohexane ring configuration known as eudesmane type (**26**–**34**), an aristolane sesquiterpenoid (**35**) and a sesquiterpene cyclized to one 10-carbon ring known as germacrane derivative (**36**), isolated majorly from the methanolic extract of the roots of *C. edulis* [8, 10]. Lindsay et al. carried out an investigation on the dichloromethane extract of the wood of *C. lanceolata* [1] and isolated carindone (**25**), carissone (**26**) and dehydrocarissone (**32**). It is noted that **25** is a sesquiterpene dimer with two eudesmane units connected by an additional ketone group. Further pharmacological test against *Staphylococcus aureus*, *Escherichia coli* and *Pseudomonas aeruginosa* indicated that all the three compounds showed activity, with carindone (**25**) and dehydrocarissone (**32**) having a minimum inhibitory concentration (MIC) less than 0.5 mg/mL against *S. aureus* and *E. coli* [1].

Galipali S. et al. investigated the anti-inflammatory potential of root methanol extracts of *C. carandas* using bioassay guided fractionation of extract based on inhibitory potential towards proinflammatory mediators (TNF-a, IL-1b and NO). They found out that carissone (**26**) exhibited potential anti-inflammatory agents as well as significant inhibition of NO production comparable to specific NO inhibitor without affecting the cell viability [56].

### Monoterpenes

**Fig. 4:**
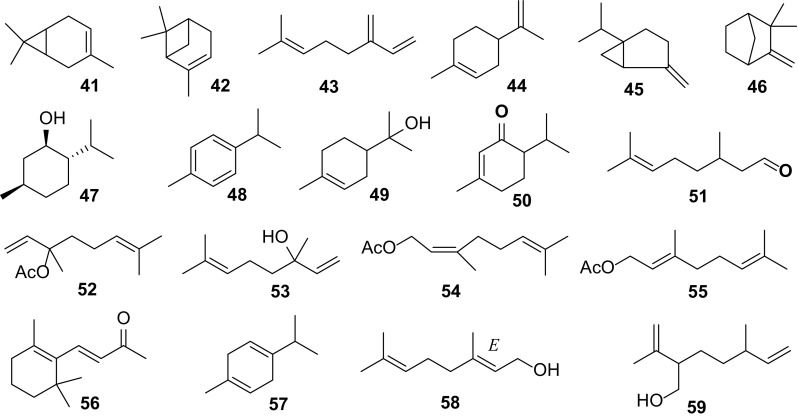
Monoterpenes **41–59** from *Carissa*

Eighteen compounds **41**–**59** (Fig. 4) have been isolated from the root oil of *C. edulis* [4] and *C. opaca* [6]. They also constitute the volatile oil of flowers of *C. carandas* and *C. grandiflora* [7].

### Flavonoids

**Fig. 5:**
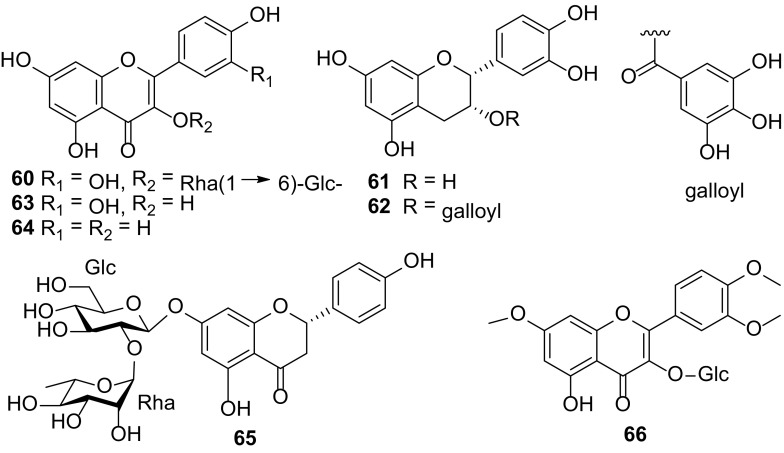
Flavonoids **60–66** from *Carissa*

This class of polyphenolic compounds occurs in any of the chemical structures such as flavones, flavonols, flavanons and flavanols. Compounds **60**–**66** (Fig. 5) have been isolated from *Carissa* species. Patil B. et al. isolated rutin (**60**), epicatechin (**61**), epicatechin gallate (**62**) and quercetin (**63**) from the berries of *C. carandas* [32]. Rutin (**60**) and kaempferol (**64**) isolated from the aerial parts of *C. edulis* displayed anti-inflammatory, arterial blood pressure and anti diuretic activities [5]. Sahreen S. et al. carried out an investigation on the fruits of *C. opaca* and reported that polyphenols and flavonoids had potent antioxidant activities in scavenging 2,2-diphenyl-1-picrylhydrazyl (DPPH), superoxides, hydroxyl, hydrogen peroxide, and ABTS radicals, and had strong iron chelating activity [81]. The ethyl acetate fraction showed the highest inhibition of *β*-carotene/linoleic acid peroxidation and phosphomolybdate assay. There were high correlations between half maximal effective concentration (EC_50_) values of DPPH, superoxide, hydroxyl, hydrogen peroxide, ABTS radical, total phenolics and flavonoids, but no significant correlation for iron chelators, *β*-carotene, and phosphomolybdate assay. Thus, the chloroform and aqueous extracts had strong antioxidant activities which correlated with high levels of polyphenols and flavonoids. These fractions may become sources of antioxidants and/or functional food ingredients.

From the literature, rutin (**60**) and quercetin (**63**) are the most predominant flavonoids in *Carissa* [5, 6, 32, 33].

### Lignans

Lignans is a class of natural products occupying quite a large portion in plants. They have been identified in about 70 families, many of which have been applied in traditional medicine. Due to their various biological effects including antimitotic, antiviral, cathartic, allergenic and antitumor activities, lignans have gained increasing attention and research interests [10]. Moreover, they are reviewed to possess antioxidant activity hence present exciting opportunities for their development as a new therapeutic base for the treatment of polygenic disorders involving oxidative stress [21].

**Fig. 6:**
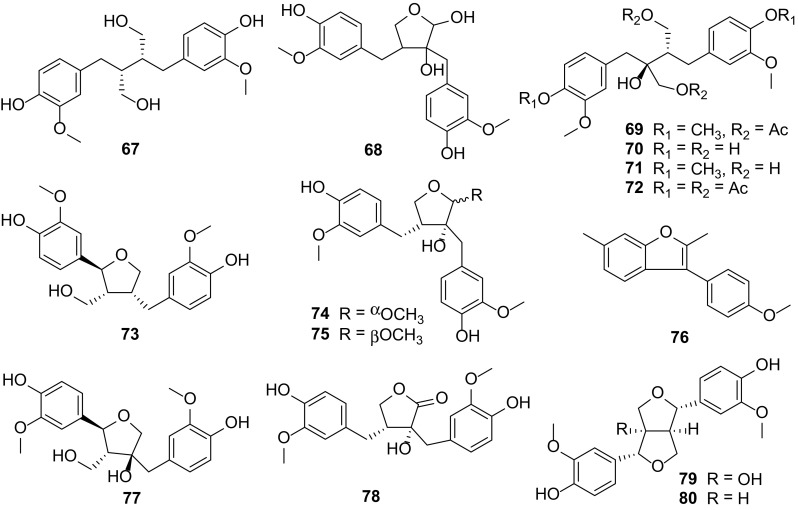
Lignans **67–80** from *Carissa*

Thirteen lignans **67**–**80** (Fig. 6) were isolated from *Carissa* species, mostly from the roots and stems as compared to the other parts of the plant. Carissanol (**68**), carinol (**70**), (+)-nortrachelogenin (**78**) and pinoresinol (**80**) were the most lignans characterized from the roots of *C. edulis* [5, 9, 21] and *C. carandas* [35], and stems of *C. lanceolata* [19] and *C. spinarum* [21, 34]. It is noted that carissanol (**68**) possessed a hemiacetal group in molecule. All three lignans carissanol (**68**), carinol (**70**) and nortrachelogenin (**79**) have shown to exhibit cytotoxicity against breast (MCF7) and lung (A549) cancer cells as well as moderate anti-DPPH free radical activity [21, 34], while (+)-nortrachelogenin (**78**) also showed antiplasmodium activity at a dose of 14.50 μg/mL [5, 9]. In addition, carinol (**70**) showed considerable antimicrobial activity against four bacteria, *P. aeruginosa*, *E. coli*, *Staphylococcus aureus* and *Bacillus subtilis*, with a MIC of <1.25 mg/mL, by a micro broth dilution technique [36].

### Sterols

**Fig. 7:**
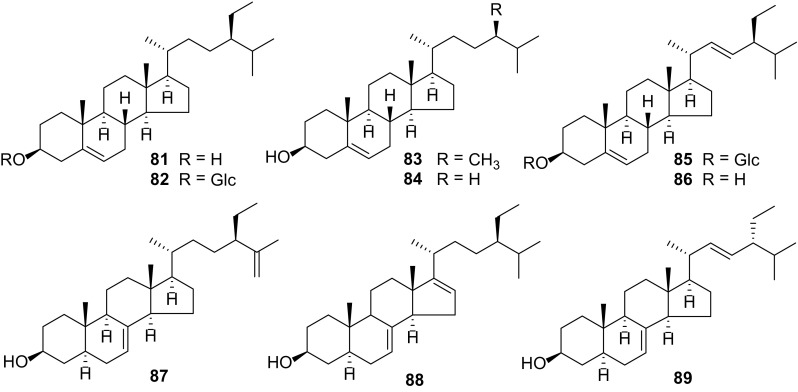
Sterols **81–89** from *Carissa*

Sterols **81**–**89** of *Carissa* (Fig. 7) have indicated possession of hepatoprotective, anti-inflammatory, anti-HIV and anti-hyperlipidemic activities [10]. They have been isolated from the roots of *C. congesta* [11, 17], *C. spinarum* [37], and *C. carandas* [15, 31, 38]. *β*-Sitosterol (**84**) is the most common sterol in *Carissa* and it is present in the leaves of *C. edulis* [5], roots of *C. opaca* [6] and *C. carandas* [15, 31, 38].

Galipali S. et al. investigated the anti-inflammatory potential of root methanol extracts of *C. carandas* using bioassay guided fractionation of extract based on inhibitory potential towards proinflammatory mediators (TNF-a, IL-1b and NO). They found out that stigmasterol (**86**) exhibited potential anti-inflammatory agents [56].

### Simple Phenolic Compounds

Phenolic compounds form the largest group of secondary metabolites produced by plants, mainly, in response to biotic or abiotic stresses such as infections, wounding, UV irradiation, exposure to ozone, pollutants and other hostile environmental conditions. They are mostly hydroxybenzoic and hydroxycinnamic acid derivatives. There has been increased interest towards natural and synthetic phenyl propanoids for medicinal use as antioxidant, UV screens, anticancer, antivirus, anti-inflammatory, wound healing and antibacterial activities [10, 55, 56].

**Fig. 8:**
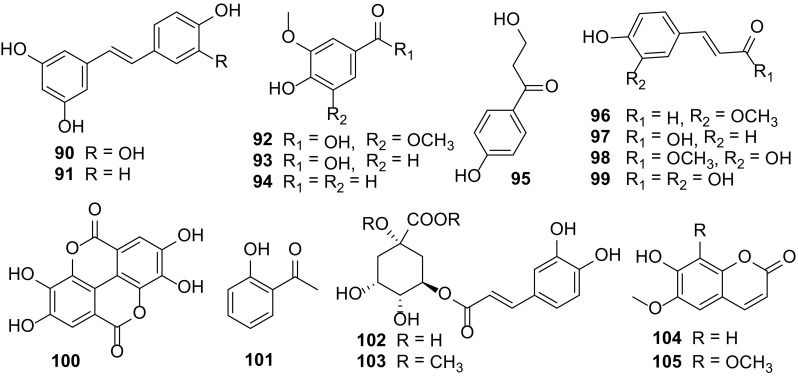
Simple phenolic compounds **90–105** from *Carissa*

Isolation of 16 phenolic compounds, **90**–**105** (Fig. 8) has been achieved from the fruits, roots, and stems of *C. carandas* [14, 32], *C. edulis* [5, 9], *C. lanceolata* [1], *C. opaca* [6, 13], and *C. spinarum* [31]. Coniferaldehyde (**96**) isolated from the stems of *C. spinarum* [31], was reported to inhibit LPS-induced apoptosis through the PKC α/β II/Nrf-2/HO-1 dependent pathway in RAW264.7 macrophage cells [82]. *p*-Coumaric acid (**97**), isolated from the fruits of *C. carandas* [32], and their derivatives had shown to exert anti-coagulant, anti-tumor, anti-viral, anti-inflammatory and antioxidant effects, as well as anti-microbial and enzyme inhibition properties [10]. Chlorogenic acid (**102**), isolated from the fruits of *C. carandas* [32], and their derivatives, possess antioxidants that might contribute to the prevention of type II diabetes mellitus, cardiovascular disease and certain aging related diseases [10].

Galipali S. et al. investigated the anti-inflammatory potential of root methanol extracts of *C. carandas* using bioassay guided fractionation of extract based on inhibitory potential towards proinflammatory mediators (TNF-a, IL-1b and NO). They found out that the coumarin, scopoletin (**104**) exhibited significant inhibition of NO production comparable to specific NO inhibitor without affecting the cell viability [56].

### Fatty Acids and Esters

**Fig. 9:**
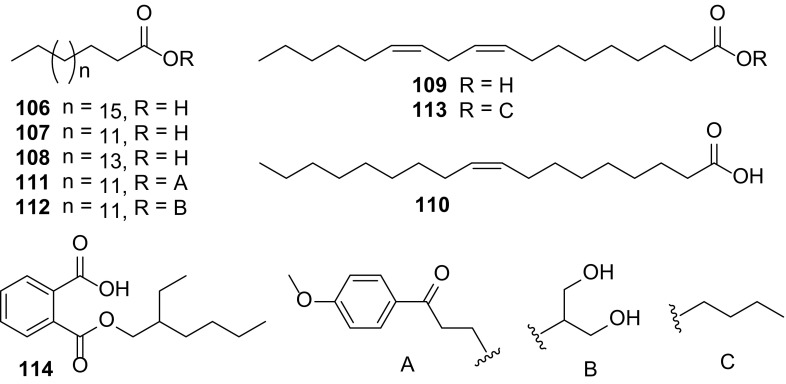
Fatty acids and esters **106–114** from *Carissa*

Nine fatty acids and esters (**106**–**114**) have been reported from the genus *Carissa* (Fig. 9). Five saturated (**106**–**108**) and unsaturated (**109**–**110**) fatty acids were isolated from the seed oils of *C. carandas* [40]. In addition, four esters, 3′-(4″-methoxyphenyl)-3′-oxo-propionyl hexadecanoate (**111**) from *C. spinarum* [21], hexadecanoic acid 2-hydroxyl-1-(hydroxymethyl) ethyl ether (**112**) and butyl-9,12-octadecadienoate (**113**) from *C. congesta* [17], and 2-benzenedicarboxylic acid mono (2-ethylhexyl) ester (**114**) from *C. opaca* [6], were also obtained.

### Others Compounds

**Fig. 10:**
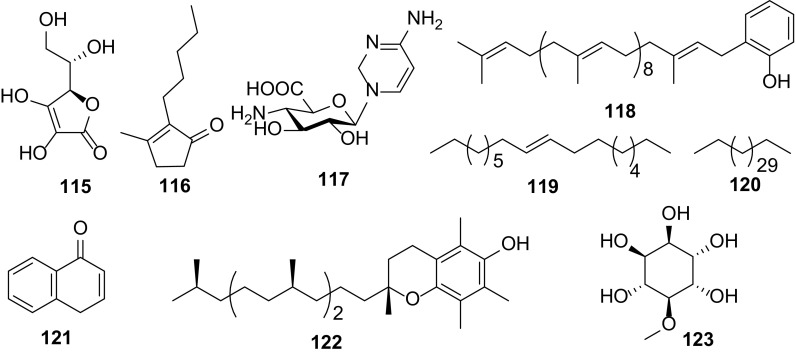
Other compounds **115–123** from *Carissa*

In addition to terpenoids, flavonoids, sterols, lignans, simple phenolics, fatty acids and esters, nine other types of compounds (**115**–**123**) have been isolated from *Carissa* species (Fig. 10). They comprise l-ascorbic acid (**115**), dihydrojasmone (**116**), 4-amino-1-(4-amino-2-oxo-1(2*H*)-pyrimidinyl)-1,4-dideoxy-β-d-glucopyranuronic acid (**117**) and 6-decaprenylphenol (**118**) from the flowers and roots of *C. carandas* [7, 32], an alkene (**119**) from the roots of *C. congesta* [17], an alkane (**120**) from the leaves of *C. bispinosa* [20], a naphthalenone (**121**) and vitamin E (**122**) from the roots of *C. opaca* [4, 6], and a cyclohexanehexol, pinitol (**123**) from the leaves of *C. edulis* [5].

## Pharmacological Studies

Researchers have investigated pharmacological activities of *Carissa* species based on their claimed ethnomedicinal and anecdotal uses, including anti-plasmodial, diuretic effect, anticonvulsant, antibacterial, antioxidants and anti-tumor, antiviral, analgesic, anti-inflammatory, antipyretic, vasorelaxant, antihypertensive, cardioprotective and hepatoprotective activities, as illustrated below.

### Anti-plasmodial Activity

Traditionally, the Meru and Kilifi communities of Kenya use the decoction of the root bark of *C. edulis* for treatment of malaria and other ailments [41]. In an investigation to determine anti-plasmodial activity of *C. edulis*, *Plasmodium falciparum* in vitro drug sensitive study was conducted in order to evaluate the correlation between the ethno medicinal use and bioactivity of the plant’s methanolic root bark extract. The extract showed anti-plasmodial activity against the chloroquin sensitive (D6) strain of *P. falciparum* parasite with minimum inhibition concentration for inhibiting 50% of the pathogen (IC_50_) value of 1.95 μg/mL. From this experiment, a lignan compound nortrachelogenin(**78**) was isolated and it showed antiplasmodium activity of 14.50 μg/mL [5].

### Diuretic Effect

It is reported that the diuretic activities of different extracts of *C. edulis* were investigated orally at a dose range of 50–1000 mg/kg in rats using hydrochlorothiazide as a standard drug. The root bark soxhlet extract produced a significant increase (*P* < 0.05) in urine output at a dose of 1000 mg/kg. The root wood maceration and root wood soxhlet extracts produced a significant increase in urine output at a dose of 50 mg/kg, with a *P* value of <0.05. Urinary electrolyte excretion was also affected by the extracts. The root bark soxhlet extract increased urinary excretion of sodium, potassium and chloride ions while the root wood maceration extract increased excretion of sodium and potassium and the root wood soxhlet extract increased excretion of potassium ion. These findings support the traditional use of *C. edulis* as a diuretic agent [42].

### Anticonvulsant Activity

In a study to investigate anticonvulsant activity of root bark extract of *C. edulis*, the median lethal dose (LD(50)) of its extract was determined using Lork’s method and the anticonvulsant activity of the extract was assessed in pentylenetetrazole—induced convulsion in mice and maximal electroshock test (MEST) in chicks, with benzodiazepine and phenytoin as standard drugs, respectively. Parallel studies were also conducted using both flumazenil, a neurotransmitter gamma-aminobutyric acid (GABA) (A)—benzodiazepine receptor complex site antagonist and naloxone a non-specific opioid receptor antagonist. The LD(50) of *C. edulis* was 282.8 mg/kg and over 5000 mg/kg following intraperitoneal and oral administration, respectively. *C. edulis* extract produced 40 and 20% protection against convulsion at 5 and 20 mg/kg, respectively, compared with 100% protection with benzodiazepine. Essentially, the mean onset and percentage protection against convulsion in *C. edulis* extract-treated mice were reduced by flumazenil and naloxone. *C. edulis* extract exhibited dose-dependent inhibition of the convulsion induced by MEST with 20 mg/kg providing 90% protection while phenytoin (20 mg/kg) produced 100% protection. The results showed that the root extract of *C. edulis* possesses biologically active constituent(s) that have anticonvulsant activity which supports the ethnomedicinal claims of the use of the plant in the management of epilepsy [43].

### Antibacterial Activity

While investigating antibacterial activity of the extracts of leaves, stems and roots of *C. carandas* using disc diffusion assay, MIC, minimum bactericidal concentration, total activity, mean and standard deviation were calculated. *Streptococcus aureus* was found to be the most susceptible organism followed by *B. subtilis* and *E. coli*. Flavonoid of roots showed the best activity against *B. subtilis* (IZ = 15 mm, MIC = 0.312 mg/mL, MBC = 0.156 mg/mL, TA = 3.20 mL/g). Results revealed that extracts of *C. carandas* have good antimicrobial potential and may be exploited for antimicrobial drugs [44]. In another study, the dichloromethane and toluene extract of the leaves of *C. carandas* showed better results against *Staphylococcus aureus* and *Klebsiella pneumonia*. The fruit extract of *C. carandas* in dichloromethane exhibited high antibacterial activity against *E. coli*. The fruit extract in ethyl acetate showed the best result against all the strains of bacteria [45]. In another investigation carried out on the root extract of *C. opaca*, the sample exhibited considerable antimicrobial activities against *B. subtilis*, *E. coli*, *P. aeruginosa*, *Candida albicans* and *Aspergillus niger* with zones of inhibition ranging from 10 to 13 mm as compared to the standard drug amoxicillin with zones of inhibition 13–17 mm under similar conditions. The roots of *C. opaca* can provide new leads for future antimicrobial drugs [13]. Further antibacterial studies on naringin(**65**) and ursolic(**6**) acid isolated from the leaves of *C. spinarum* had similar antibacterial activities and they completely inhibited the pathogenic Gram negative bacteria which causes diarrhea and dysentery [19].

### Antioxidants and Anti-tumor Activity

While carrying out cytotoxicity investigation on *C*. *carandas* extracts against cancer cell lines, the compound carandinol (**21**) from the leaves of *C*. *carandas* [24] exhibited significant in vitro cytotoxicity to every cell line tested (HeLa, PC-3 and 3T3) and was relatively more toxic to human cervical cancer (HeLa) cell line [24]. Lignans carissanol (**68**), carinol (**70**) and nortrachelogenin (**78**), from the stems of *C. spinarum* [34] have been shown to exhibit cytotoxicity against breast (MCF7) and lung (A549) cancer cells. Moreover, moderate anti-DPPH free radical activity has been observed for all the lignans [21, 46]. In a different study, *C. spinarum* aqueous extract and its *n*-butanol fraction exhibited potential cytotoxic effect on a wide range of human cancer cell lines, with apoptotic activity in human leukaemia HL-60 cells through the mitochondrial dependent pathway in HL-60 cells [47]. In another investigation to determine the antioxidant and DNA damage inhibition potential of leaf methanolic extract of *C. carandas*, the extract had significant (*P* < 0.05), dose-dependent DPPH radical scavenging activity (median inhibitory concentration 73.1 µg/mL), total antioxidant activity, H_2_O_2_ scavenging activity (median inhibitory concentration 84.03 µg/mL) and reducing power activity. It was also found out that the extract completely protected pBR 322 plasmid DNA from free radical-mediated oxidative stress in a DNA damage inhibition assay. The antioxidant and DNA damage inhibition properties of *C. carandas* can be attributed to a high content of phenolic compounds (84.0 mg gallic acid equivalents/g dry weight of extract). The high antioxidant and DNA damage inhibiting potential of *C. carandas* could be used to develop antioxidant compounds for therapeutic applications [48]. Further cytotoxicity investigations have been performed on *C. opaca* extracts. In one of such studies, *C. opaca* extracts and fractions were tested against MCF7 breast cancer cell line using 3-(4,5-dimethylthiazol-2-yl)-2,5-diphenyl tetrazolium bromide (MTT) assay, a concentration dependent inhibition of 78.5% activity was observed for the crude extracts against cancer cells at a concentration of 500 μg/mL. Fractions were tested at a concentration of 200 μg/mL and were more active than crude extracts. Chloroform fraction showed maximum inhibition of 99% followed by ethyl acetate and methanol fraction exhibiting 96 and 94% inhibition, respectively [49].

### Antiviral Activity

In a certain investigation, an aqueous total extract preparation of the roots of *C. edulis* exhibited remarkable anti-HSVs activity in vitro and in vivo for both wild type and resistant strains of HSV. The extract significantly inhibited formation of plaques in Vero E6 cells infected with 100 plaque forming units of wild type strains of HSV (7401 H HSV-1 and Ito -1262 HSV-2) or resistant strains of HSV (TK (-) 7401H HSV-1 and AP (r) 7401H HSV-1) by 100% at 50 mg/mL in vitro with minimal cell cytotoxicity (CC_50_ = 480 mg/mL). When the extract was examined for in vivo efficacy in a murine model using Balb/C mice infected with wild type or resistant strains of HSV, the extract, at an oral dose of 250 mg/kg, significantly delayed the onset of HSV infections by over 50%. It also increased the mean survival time of treated infected mice by between 28 and 35% relative to the infected untreated mice (*P* < 0.05 vs. control by Student’s t-test). The mortality rate for mice treated with extract was also significantly reduced by between 70 and 90% as compared with the infected untreated mice that exhibited 100% mortality. No acute toxicity was observed in mice at the oral therapeutic dose of 250 mg/kg. These results suggest that the root aqueous extract of *C. edulis* contain potent anti-viral agents against HSVs that can be exploited for development of an alternative remedy for HSV infections [50, 51]. A separate investigation on the hexane extract of *C. edulis* displayed moderate activity against feline herpes virus 1 with EC_50_ <70 mg/mL and SI value <2. On the other hand, excellent activity was exhibited with the hexane extracts of *C. edulis* against canine distemper virus [52].

### Antiemetic Activity

A study conducted to explore the antiemetic activity of the fruit ethanol extract of *C. carandas* using chick emetic model showed a decrease in retches induced by copper sulfate pentahydrate given orally at 50 mg/kg body weight [53].

### Anti-hyperlipidemic Activity

The lipid lowering activity of aqueous: ethanol (1:1) extract of *C. carandas* in egg yolk induced hyperlipidemic rats showed a highly significant increase in the weight of high cholesterol diet rats. The extract caused a significant reduction in body weight, cholesterol, triglycerides, HDL and LDL in hyperlipidemic rats. Histopathological changes induced by high cholesterol diet were also significantly reduced by the extract. The activity of the extract of *C. carandas* at a dose of 1000 mg/kg was comparable to that of atorvastatin at a dose of 0.2 mg/kg [54].

### Analgesic, Anti-inflammatory and Antipyretic Activities

Analgesic activity of *C. carandas* was studied in mice using hot plate and acetic acid induced writhing methods, while carrageenan induced paw edema was used to access anti-inflammatory activity. The antipyretic activity was evaluated by Brewer’s yeast induced pyrexia in rats. Ethanol and aqueous extracts from roots of *C. carandas* exhibited significant (*P* < 0.01) analgesic, anti-inflammatory and antipyretic activities at doses of 100 and 200 mg/kg body weight. In analgesic activity, the highest percentage of inhibition of abdominal constriction (72.67%) was observed for ethanol extracts of *C. carandas* at a dose of 100 mg/kg body weight. The ethanol and aqueous extracts from *C. carandas* were found to reduce significantly the formation of edema induced by carrageenan after 2 h. Both the extracts of *C. carandas* showed significant antipyretic activities on yeast induced hyperpyrexia in rats after 2 h. The results of this study indicated that the ethanol and aqueous extracts from the roots of *C. carandas* possess significant analgesic, anti-inflammatory and antipyretic activities in rodent models [55]. In a separate study to investigate the anti-inflammatory potential of root methanol extracts of *C. carandas* involving bioassay guided fractionation of extract based on inhibitory potential towards proinflammatory mediators (TNF-a, IL-1b and NO), it was found out that lupeol (**1**), oleanolic acid (**15)**, carissone (**26**), stigmasterol (**86**), and scopoletin (**104**) possess potential anti-inflammatory agents. Carissone (**26**) and scopoletin (**104**) exhibited significant inhibition of NO production comparable to specific NO inhibitor without affecting the cell viability [56]. *C. spinarum* has been used traditionally for the treatment of inflammation-related disorders such as rheumatic pain and to relieve fever. Based on this information, ethanolic extract of the roots of *C. spinarum* was evaluated for its antipyretic activity. Wistar albino rats were induced with Brewer’s yeast (2 mL/kg) for pyrexia and antipyretic activity was assessed with 100, 200 and 400 mg/kg ethanolic extract. The ethanolic extract significantly (*P* < 0.05) reduced the elevated body temperature in a dose dependent manner [57].

### Vasorelaxant and Antihypertensive Activities

In an effort to investigate vasorelaxant activity of the leaves of *C. spinarum* extracts as a possible source of compounds with antihypertensive effects, an experiment was carried out using an ex vivo method. All tested extracts caused concentration-dependant relaxation in pre-contracted aortic rings. The dichloromethane soluble extracts from the leaves of *C. spinarum* was the most active (EC_50_ = 0.17 ± 0.01 mg/mL, E_max_ = 85.72%). The ability of the extracts in this study to cause relaxation of the aortic rings pre-contracted with phenylephrine could rationally explain the use of *C. spinarum* to treat hypertension by Malagasy traditional healers in Madagascar [58].

### Cardioprotective

The protective effects of various fractions of leaf extract of *C. opaca* against carbon tetrachloride (CCl_4_) administration was reviewed by rat cardiac functions alterations. Chronic toxicity caused by 8 week treatment of CCl_4_ to the rats significantly changed the cardiac function test, decreased the activities of antioxidant enzymes and glutathione contents whereas significant increase was found in lipid peroxidation. Administration of various fractions of the extract with CCl_4_ showed protective ability against CCl_4_ intoxication by restoring the cardiac functions alterations, activities of antioxidant enzymes and lipid peroxidation in rat. CCl_4_ induction in rats also caused DNA fragmentation and histopathological abnormalities which were restored by administration of various fraction of *C. opaca* leaves extract. Results revealed that various fraction of *C. opaca* are possess in cardio-protective agents [59].

### Hepatoprotective Activity

Ethyl acetate fraction of the ethanol extract from roots of *C. carandas* was studied against CCl_4_-, paracetamol-, and ethanol-induced hepatotoxicity in rats. Significant hepatoprotective effects were obtained against liver damage induced by all the three toxins, as indicated by changed biochemical parameters like serum transaminases, alkaline phosphate, total bilirubin, total protein, and total cholesterol. The ethyl acetate fraction prevented toxin-induced oxidative stress by significantly maintaining the levels of reduced glutathione and malondialdehyde, and a normal functioning of the liver, compared to toxin controls [38]. *C. carandas* root extract is used by tribal healers of Western Ghat region of Karnataka as hepatoprotective and antihyperglycemic [60]. A study was conducted to evaluate the hepatoprotective effects of the ethanol and aqueous extracts of roots of *C. carandas* against ethanol induced hepatotoxicity in rats. Their liver function test, serum lipid profile, levels of lipid peroxidation and the activity of liver antioxidant enzyme glutathione were established at a dose level of 100 and 200 mg/kg. The effect produced significant hepatoprotection by decreasing serum transaminase bilirubin and lipid peroxidation, while it significantly increased the levels of liver glutathione and serum protein [61]. In another similar investigation, oral pre-treatment with ethanolic extract of the roots of *C. carandas* showed significant hepatoprotective activity against CCl_4_ and paracetamol induced hepatotoxicity by decreasing the activities of serum marker enzymes, bilirubin and lipid peroxidation, and significant increase in the levels of uric acid, glutathione, super oxide dismutase, catalase and protein in a dose dependent manner, which was confirmed by the decrease in the total weight of the liver [62].

### Other Effects

Oral administration of the ethanolic extracts of the leaves of *C. edulis* on blood glucose levels both in normal and streptozotocin (STZ) diabetic rats significantly reduced the blood glucose level in STZ diabetic rats during the first 3 h of treatment [63]. The roots of *C. carandas* are used in the treatment of helminthiasis [64] Tannins from the leaves of *C. spinarum* possess antihelminthic properties [65].

A study conducted to investigate inhibitory activities of the methanolic extract, ethyl acetate and chloroform, aqueous and hexane fractions of *C. opaca* roots against xanthine oxidase (XO) and alpha-amylase enzymes showed significant results. Methanolic extract displayed significant activity against both the enzymes with IC_50_ of 156.0 and 5.6 mg/mL for XO and alpha-amylase, respectively. Ethyl acetate fraction showed highest activity against both the enzymes with IC_50_ of 129 and 4.9 mg/mL for XO and alpha-amylase, respectively. Chloroform fraction had IC_50_ of 154.2 and 5.5 mg/mL for XO and alpha-amylase, respectively. Aqueous fraction exhibited significant efficacy against alpha-amylase (IC_50_ 5.0 mg/mL). Hexane fraction showed good activity against alpha-amylase in a dose-dependent manner but exhibited opposite trend against XO [6].

Crude extract of *C. carandas* possesses laxative and antidiarrheal properties mediated through combinations of gut stimulant and inhibitory activities. The gut stimulant potential of *C. carandas* was found mediated through combination of muscarinic and histaminergic receptors activation, while its gut inhibitory activity was observed mediated through antagonistic pathway. This study provides a rationale for the medicinal use of *C. carandas* in constipation and diarrhea [67]. The roots of *C. carandas* and *C. spinarum* are used as a purgative and as an antidote for snakebite, and the leaves for remittent fever [68]. In vitro inhibitory activity of leaf extracts of *C. spinarum* in non-polar and polar solvents was determined against *Bungarus caeruleus* and *Vipera russelli* toxic snake venom enzymes. Methanol extracts (100 μg/mL) inhibited acetylcholinesterase, phospholipase A_2_, hyaluronidase, phosphomonoesterase, phosphodiesterase, 5′-nucleotidase enzymes of *B. caeruleus* and *V. russelli* venoms [69].

Ethanol extract from the roots of *C. spinarum* has exhibited ability to lower blood pressure in cats [77]. The in vivo wound healing activity of 1 and 2.5% (w/w) *C. spinarum* extract was assessed on a burn wound model in mice by the rate of wound contraction, period of epithelization and hydroxyproline content and the results showed that *C. spinarum* root extract has significant wound healing activity as evident from the rate of wound contraction and epithelisation [77].

In the management of chronic joint pains in Machakos County of Kenya, the leaves, stems and roots of *C. spinarum* are boiled in water and concoction drunk with soup, one glass three times daily, for 14 days or until a patient recovers [79].

Sahreen S. et al. carried out an investigation on the fruits of *C. opaca* and reported that polyphenols and flavonoids had potent antioxidant activities in scavenging DPPH, superoxides, hydroxyl, hydrogen peroxide, and ABTS radicals, and had strong iron chelating activity [81].

## Conclusion and Future Prospects

From this review, it can be deduced that the major compounds of *Carissa* are terpenes, lignans and simple phenolic compounds. Amongst terpenes and lignans, compounds such as carandinol (**21**) and nortrachelogenin (**78**) have exhibited anti-tumor activity. The review highlights that compounds from fruits, leaves and roots of *Carissa* not only contain biological properties but have also exhibited significant biological activities such as antitumor, antibacterial, antiplasmodial, antiviral, anti-hyperlipidemic, amongst others. It would therefore be important to extensively investigate their phytochemicals and pharmacologically determine their activities for future drug discovery and development. *Carissa* seems to possess great potential, yet majority of its species’ chemical constituents remain unknown. It would be very necessary for the pharmacology community to explore and investigate more of its species in order to determine their chemical constituents and report their potential.

## Abbreviations

IC_50_: Minimum inhibition concentration for inhibiting 50% of the pathogen
CC_50_: Cytotoxic concentration of the extracts to cause death to 50% of host’s viable cells
EC_50_: Half maximal effective concentration
MIC: Minimum inhibitory concentration
GABA: Neurotransmitter gamma-aminobutyric acid
DPPH: 2,2-Diphenyl-1-picrylhydrazyl
MTT: 3-(4,5-Dimethylthiazol-2-yl)-2,5-diphenyl tetrazolium bromide
